# Association of physical activity with utilization of long-term care in community-dwelling older adults in Germany: results from the population-based KORA-Age observational study

**DOI:** 10.1186/s12966-022-01322-z

**Published:** 2022-08-08

**Authors:** Kathrin Steinbeisser, Larissa Schwarzkopf, Lars Schwettmann, Michael Laxy, Eva Grill, Christian Rester, Annette Peters, Hildegard Seidl

**Affiliations:** 1 Institute of Health Economics and Health Care Management, Helmholtz Zentrum München, Ingolstädter Landstraße 1, 85764 Neuherberg, Germany; 2 Faculty for Applied Healthcare Sciences, Technical University of Deggendorf, Dieter-Görlitz-Platz 1, 94469 Deggendorf, Germany; 3 IFT Institut für Therapieforschung, Leopoldstr. 175, 80804 Munich, Germany; 4 Department of Economics, Martin Luther University Halle-Wittenberg, 06099 Halle (Saale), Germany; 5 TUM Department of Sport and Health Sciences, Professorship of Public Health and Prevention, Technical University of Munich, Georg-Brauchle-Ring 60/62, 80992 Munich, Germany; 6 School of Public Health, Emory University, 1518 Clifton Rd, Atlanta, GA 30322 USA; 7 German Center of Diabetes Research (DZD), Munich, Germany; 8 Institute for Medical Information Processing, Biometry and Epidemiology (IBE), Ludwig-Maximilians-University Munich, Marchioninistr. 15, 81377 Munich, Germany; 9 German Center for Vertigo and Balance Disorders, Ludwig-Maximilians-University Munich, Marchioninistr. 15, 81377 Munich, Germany; 10 Institute of Epidemiology, Helmholtz Zentrum München, German Research Center for Environmental Health, Ingolstädter Landstraße 1, 85764 Neuherberg, Germany; 11 Quality Management and Gender Medicine, München Klinik Schwabing, Kölner Platz 1, 80804 Munich, Germany

**Keywords:** Sports, Health care utilization, Nursing care, Elderly, Gender, Prevention, Health promotion, Active lifestyle, Generalized estimating equations, National guidelines

## Abstract

**Background:**

Physical activity (PA) is a proven strategy to prevent chronic diseases and reduce falls. Furthermore, it improves or at least maintains performance of activities of daily living, and thus fosters an independent lifestyle in older adults. However, evidence on the association of PA with relevant subgroups, such as older adults with utilization of long-term care (LTC), is sparse. This knowledge would be essential for establishing effective, need-based strategies to minimize the burden on healthcare systems due to the increasing need for LTC in old age.

**Methods:**

Data originate from the 2011/12 (t_1_) baseline assessment and 2016 (t_2_) follow-up of the population-based Cooperative Health Research in the Region of Augsburg (KORA-)Age study in southern Germany. In 4812 observations of individuals ≥65 years, the association between various types of PA (walking, exercise (i. e., subcategory of PA with the objective to improve or maintain one or more components of physical fitness), walking+exercise) and utilization of LTC (yes/no) was analyzed using generalized estimating equation logistic models. Corresponding models stratified by sex (females: 2499 observations; males: 2313 observations) examined sex-specific associations. Descriptive analyses assessed the proportion of individuals meeting the suggested minimum values in the German National Physical Activity Recommendations for older adults (GNPAR).

**Results:**

All types of PA showed a statistically significant association with non-utilization of LTC in the entire cohort. “Walking+exercise” had the strongest association with non-utilization of LTC in the entire cohort (odds ratio (OR): 0.52, 95% confidence interval (CI): 0.39–0.70) and in males (OR: 0.41, CI: 0.26–0.65), whereas in females it was “exercise” (OR: 0.58; CI: 0.35–0.94). The proportion of individuals meeting the GNPAR was higher among those without utilization of LTC (32.7%) than among those with LTC (11.7%) and group differences were statistically significant (*p* ≤ 0.05).

**Conclusions:**

The GNPAR are rarely met by older adults. However, doing any type of PA is associated with non-utilization of LTC in community-dwelling older adults. Therefore, older adults should be encouraged to walk or exercise regularly. Furthermore, future PA programs should consider target-groups’ particularities to reach individuals with the highest needs for support.

**Supplementary Information:**

The online version contains supplementary material available at 10.1186/s12966-022-01322-z.

## Background

The beneficial effects of regular physical activity (PA) on older adults’ physical, psychological, and social well-being have been shown in various systematic reviews [1–3]. Furthermore, PA is a proven strategy to promote health, prevent chronic diseases, and reduce falls. It also improves or at least maintains performance of activities of daily living, and thus fosters an independent lifestyle in older adults [4]. Despite PA’s health benefits, older adults rarely follow the World Health Organization’s (WHO’s) recommendation of 150 minutes of moderate PA (on a scale relative to an individual’s personal capacity between 0 and 10: usually 5 or 6) per week or 75 minutes of vigorous PA (rating: usually 7 or 8) per week [5–8]. Most high-income countries report that 20–60% of adults ≥65 years follow WHO’s recommendations [7, 8]. Regarding PA patterns (e. g., duration, type, frequency) and PA’s effects, differences between sexes should also be considered [8–12]. For example, females are less likely to do PA regularly than males [8]. Also, older males tend to do more vigorous exercise than older females [9].

The current evidence about PA’s health benefits for older adults and particularities of PA in relevant subpopulations (e. g., sexes), as well as the low proportion of older adults meeting WHO’s recommendations, are important to consider as populations age worldwide. By 2030, one in six people will be 60 years of age or older [13]. This trend is linked to an increasing burden on health care systems caused by older adults’ considerable need for health care and long-term care (LTC) services [13]. The increasing demand for LTC services in old age is one of the main cost drivers in health care; thus, it is advisable that politicians and public health professionals seek out potentially effective strategies, such as PA interventions, to reduce the need for LTC services in old age [14].

WHO states in its “Guidelines on physical activity and sedentary behaviour” that notable gaps in evidence regarding the behavior of specific subpopulations remain, thus inhibiting the development of target-oriented programs for them [5]. In old age, community-dwelling older adults with and without LTC make up a large proportion of the entire population, and are thus a highly considerable subpopulation in our societies [13]. However, comprehensive evidence about the role of PA with respect to utilization of LTC in this subpopulation is sparse. Furthermore, deeper knowledge about the implications of distinct types of PA, which, in old age, might be, e. g., walking or exercise, as well as the consideration of sex-specific particularities in regard to utilization of LTC, is lacking [15–18]. Additionally, detailed analyses comparing older community-dwelling adults with and without utilization of LTC meeting PA recommendations are still missing. This inhibits the assessment of this subpopulation’s vulnerability and the benefits of promoting PA in this group.

In light of the existing evidence and its gaps, it is highly important to gain further knowledge about PA, its implications on utilization of LTC in relevant subpopulations like community-dwelling older adults, and subpopulation particularities. This information would enable policy-makers to identify vulnerable target groups and set up need-based PA interventions, whose effects could mitigate the growing public health problem of increasing demand for LTC services.

To contribute to closing the existing research gaps, this study has the following objectives: 1) to determine the association of PA with utilization of LTC in community-dwelling older adults; 2) to detect differences regarding the sex-specific association of PA with utilization of LTC in females and males; 3) to determine the proportion of community-dwelling older adults with and without utilization of LTC meeting the suggested minimum values for distinct types of PA according to the “German National Physical Activity Recommendations” for older adults (GNPAR).

## Methods

### Study population

We used data from the Cooperative Health Research in the Region of Augsburg (KORA)-Age study, which is a part of the regional KORA research platform for population-based health research in Germany. The KORA research platform consists of population-based surveys and their follow-up studies. The KORA-Age study is a follow-up of participants ≥65 years from four independent cross-sectional samples who completed health surveys conducted between 1984 and 2001 [19]. A population-representative selection of participants from population registries in the city of Augsburg along with two adjacent counties (total population in 2016: 668,500) in the federal state of Bavaria took place [20].

At Age1 (t_0_, in 2008), the eligible study population consisted of 5986 individuals born before 1944. The first follow-up, Age2 (t_1_, in 2011/12), only included individuals from a sex- and age-stratified subsample of Age1 participants (*n* = 1079) with 100 people per stratum (males and females in five age groups, i. e. ten strata). Of those, 822 participated in medical examinations and completed a telephone interview (response rate: 84.3%) consisting of validated questions on, e. g., sociodemographic characteristics, PA, morbidity, and utilization of health care services [21]. Proxies (e. g., informal caregivers) were interviewed if the participant was unable to answer the questions (*n* = 29 [3.5% of participants]). For the second follow-up (Age3, t_2_, in 2016), the total sample of Age1 and individuals from the four cross-sectional samples who were born before 1951, and thus aged ≥65 years in 2016, were invited to participate. This resulted in an eligible study population of 6051 at Age3 (t_2_). Of those, 4083 participated in telephone interviews and questionnaires (response rate: 67.5%; completed by proxies: *n* = 191 [4.7% of participants]).

**Fig. 1:**
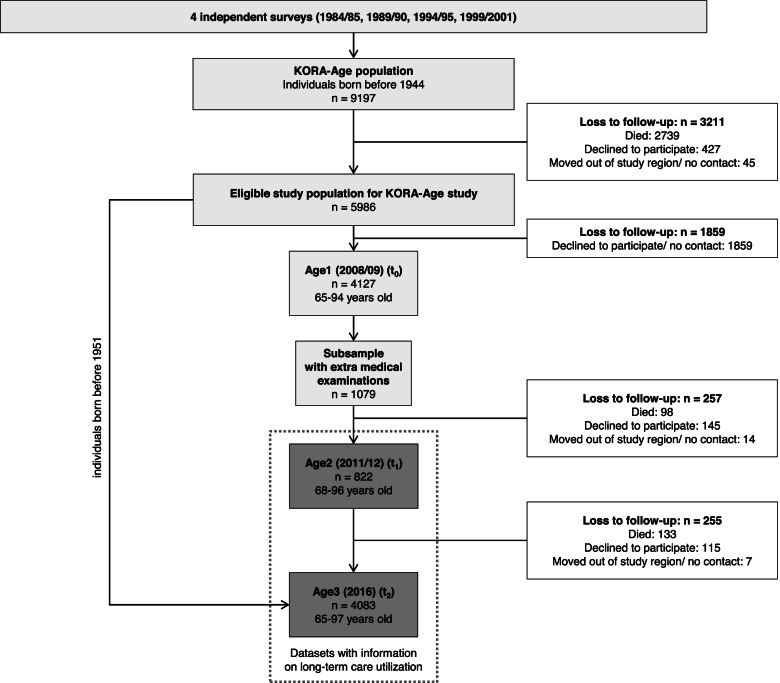
Flow chart for KORA-Age population

Since Age1 (t_0_) did not assess information on utilization of LTC, only Age2 (t_1_) and Age3 (t_2_) were considered for analyses. For the main analysis, we used individuals from t_1_ (*n* = 822) and t_2_ (*n* = 4083) [see Fig. 1].

Approval for the KORA-Age study was obtained from the Ethics Committee of the Bavarian Medical Association. Individuals agreed to participation with informed consent. Further details on data collection, study design, sampling, and response rates are described elsewhere [22, 23].

### Measurement and operationalization of physical activity

According to the World Health Organization (WHO), PA is defined as “any bodily movement produced by skeletal muscles that requires energy expenditure” [5]. PA is an umbrella term for different forms of movement [24]. In this study, we investigated “exercise” and “walking” as types of PA. In the following lines, relevant information about PA according to the “Physical Activity Questionnaire Reporting Checklist” from Nigg et al. is reported [24].

“Exercise” is a planned, repetitive, purposeful, and structured subcategory of PA with the objective to improve or maintain one or more components of physical fitness [5, 7]. In our study, it was assessed with these two questions addressing duration of exercise: “How often do you exercise during winter?” and “How often do you exercise during summer?”. Response categories for those questions were (1) “regularly more than or equal to two hours per week”; (2) “regularly more than or equal to one, but less than two hours per week”; (3) “less than one hour per week”; and (4) “no exercise”. As operationalized by Karl et al. [25], the two responses for summer and winter were initially combined as one variable with the values “high exercise” (= (1) in both summer and winter), “moderate exercise” (= combinations for summer and winter of “(1) + (2)”, “(2) + (2)”, or “(1) + (3)”), “low exercise” (= combinations for summer and winter of “(1) + (4)”, “(2) + (3)”, “(2) + (4)”), and “no exercise” (= combinations for summer and winter of “(3) + (3)”, “(3) + (4), “(4) + (4)”). To facilitate the analyses and interpretation of results, we further dichotomized these values into “high or moderate exercise” (hereafter called “exercise”) and “no or low exercise” (hereafter called “no/low exercise”). Additional file 1 illustrates a detailed differentiation of the categories.

“Walking” was assessed with the following question addressing duration of walking: “On a typical weekday, how much time do you spend walking? For example, going for a walk, on the way to work or shopping?”. Possible response categories were (1) “more than or equal to one hour”; (2) “more than or equal to half an hour, but less than one hour”; (3) “more than or equal to a quarter of an hour, but less than half an hour”; (4) “less than a quarter of an hour”; or (5) “not applicable” (= no walking due to, e. g., using a wheelchair). To facilitate the analyses and interpretation of results, we further dichotomized these values into “high or moderate walking” (= (1) or (2), in the following: “walking”) and “no or low walking” (= (3), (4), or (5), in the following: “no/low walking”). “Walking+exercise” was applied to individuals doing both “exercise” and “walking”.

The domain of PA – with the exception of walking while “going to work” representing occupational- or transport-based PA – was mainly leisure-time [24]. However, in Germany most people ≥65 years are retired, which allows focusing on “leisure-time PA”. Recall periods were “a typical (summer/winter) season” (exercise), and “a typical weekday” (walking) [26].

To address important components of PA, frequency (number of sessions per week), intensity (walking, moderate-intensity exercise, vigorous-intensity exercise, strength training), and time (average duration of an individual session per week; unit of measurement: “minutes per week”) were assessed in the subpopulation at t_2_. Questions, response options, and the calculation of the amount of PA per week can be found in Additional file 2.

### Measurement of utilization of long-term care

LTC is defined as support with daily activities for people who experience decline in self-care on a long-term basis (> three months) [27]. Daily activities consist of activities of daily living (ADLs) (e. g., dressing, bathing) and instrumental activities of daily living (IADLs) (e. g., cooking, cleaning) [28]. In Germany, there are three main forms of assistance in LTC for community-dwelling adults: formal support with ADLs, formal support with IADLs, and informal support with both ADLs and IADLs [29, 30]. Study participants were asked if they had received LTC due to their health status within the last three months [21]. Types could be (1) a home nursing service (i. e., formal support with ADLs); and/or (2) paid services for household support (i. e., formal support with IADLs); and/or (3) support from friends, family members, or neighbors (i. e., informal support with both ADLs and IADLs). If participants answered “yes” for at least one of the three types, the variable “utilization of LTC” (yes/no) was coded as “yes”. As all individuals were community-dwelling, settings for LTC were either community- or home-based.

### Covariates

Covariates related to LTC and PA were identified based on Andersen’s Behavioral Model of Health Services Use (ABMHS) [18, 31–34], the GNPAR [35], and common correlates of PA in adults [36].

Identified sociodemographic factors were age, sex, education, living arrangement, and income. Age was the participant’s age on the interview date. Sex was defined as the biological distinction of “female” or “male”. Education was comprised of school education, education at university, and vocational training. It was expressed in years (8–17 years). Living arrangement was divided into living “alone” or “not alone”. Income was defined as self-perceived income sufficiency (subjective income). In older adults, this is a common approach to express individuals’ personal evaluations of the relationship between wealth or objective income and their expenses [37]. Participants were asked if, on average, their income was enough to support them until the end of the month. The responses were divided into “sufficient” or “scarce/insufficient”.

Identified health-related factors were falls, multimorbidity, disability score, and Body Mass Index (BMI). Falls were reported as “≥ 1 fall” or “no falls/unknown” within the last year. We used participant self-reports to calculate their Charlson Comorbidity Index [38]. The index considers 13 types of chronic conditions: lung, heart, joint, kidney, gastrointestinal, liver, neurological, and eye diseases; stroke; diabetes mellitus; cancer; hypertension; and HIV [39]. In our study, multimorbidity was defined as the sum of reported chronic conditions ranging from 0 to 13. The Stanford Health Assessment Questionnaire Disability Index (HAQ-DI) was used to measure disability [40]. The HAQ-DI analyzes impairments in IADLs and ADLs. It consists of 20 questions about physical function in eight domains: dressing and grooming, hygiene, eating, standing up, walking, reach, grip, and activities [40]. Its responses range from 0 (no difficulty) to 3 (unable to perform). The highest score in a domain was taken as the domain’s score. The mean of all eight domains constituted the HAQ-DI, which was reported as a continuous value. Participants’ height and weight were measured at the study center through consistent and validated measurement methods (daily calibrated scales; stadiometer) at t_1_ and were assessed using self-reports following detailed instructions from trained telephone-interviewers at t_2_. From these values, their BMI in kg/m^2^ was calculated.

### Statistical analyses

We assessed participants’ characteristics at both follow-up timepoints, dropouts, PA values, and comparisons with the GNPAR using descriptive statistics. To investigate the association of different types of PA (walking, exercise, walking+exercise) with utilization of LTC at t_1_ or t_2_ (i. e., cross-sectional analysis with repeated measurements), we applied a generalized estimating equation (GEE) logistic model with an unstructured correlation matrix. The model accounts for repeated measurements and their intra-subject correlation [41]. As the study focused on population-averaged effects and not individual, subject-specific changes, we did not apply mixed models, which would have been an alternative for examining intra-subject correlation [42].

In the first step, we analyzed the association of distinct types of PA with utilization of LTC in the entire cohort (*n* = 4812 observations: sum of t_1_ (*n* = 800) and t_2_ (*n* = 4012)). As the existence of differences between sexes for utilization of health care services and lifestyle habits is well-known [17, 18, 34], in the second step we applied two sex-stratified models: one for females (*n* = 2499 observations: t_1_: *n* = 395, t_2_: *n* = 2104) and one for males (*n* = 2313 observations: t_1_: *n* = 405, t_2_: *n* = 1908). As disability (expressed as, e. g., “poor health” or “impaired general physical functioning” [43–46]) is one of the major barriers to PA, we applied the sensitivity analysis (SA) SA1 to each model (entire cohort, females, males). The SA1 included only observations without disability, defined as an HAQ-DI < 0.5 [47, 48]. For the general model (entire cohort) and the observations without disability (SA1), we assessed the interaction of sex with types of PA through the calculation of the respective interaction terms.

In all models, we compared observations with utilization of LTC to those without utilization of LTC. The covariates sex and education did not change from t_1_ to t_2_ and thus were considered fixed variables. All other covariates were treated as time-dependent.

Individuals with either missing values in all types of LTC (= transformation variable “utilization of LTC” (outcome)) or in both “exercise” and “walking” (= transformation variable “PA” (exposure)) were excluded through listwise deletion [49, 50]. This resulted in a final sample size of *n* = 800 at t_1_ and *n* = 4012 at t_2_. Missing values in subdomains of the variables “utilization of LTC” (t_2_: home nursing service and paid services for household support (*n* = 1); assistance of family members, friends, or neighbors (n = 1)) or “PA” (t_1_: walking (*n* = 2); t_2_: exercise (*n* = 2); walking (n = 8)) were imputed through single stochastic regression imputation using logistic regression with the fully conditional specification method [51]. This imputation strategy is based on the assumption that missing values are missing at random, meaning that they are conditionally independent from the unobserved value, hence the underlying missing data pattern is arbitrary [52, 53]. To test our model’s robustness, we conducted SA2. It excluded observations with missing values in the above-mentioned subdomains of outcome or exposure.

Regarding covariates, twenty missing values (2.5%) at t_1_ (multimorbidity (*n* = 9), BMI (*n* = 7), income (*n* = 4)), and 126 missing values (3.1%) at t_2_ (BMI (*n* = 53), income (*n* = 35), multimorbidity (*n* = 27), falls (n = 5), disability score (n = 2), living arrangement (n = 2), education (n = 2)) were identified. We imputed binary variables using single stochastic regression with the fully conditional specification method and continuous variables using predictive mean matching [51]. We based imputation of all missing values mainly on the models’ covariates (|correlation coefficient| > 0.4) [49].

We tested for multicollinearity of covariates in all models (threshold: |r| ≤ 0.8). We calculated odds ratios (OR) and 95% confidence intervals (CI). In all analyses, results with *p*-values ≤0.05 were considered statistically significant. We performed all statistical analyses using SAS software, release 9.4 (SAS Institute, Cary, NC).

## Results

### Characteristics of study sample

Table 1 characterizes the total study sample and the sample stratified by utilization of LTC and type of PA at t_2_. Out of 4012 individuals, 762 (19.0%) received LTC. Age in the entire cohort ranged from 65 to 97 years with a mean age of 75.0 years (standard deviation (SD): 6.6). The entire cohort (with missings) included 2099 females (52.5%), with 478 (22.8%) of them receiving LTC. Out of 1903 males, 276 received LTC (14.5%). In individuals without utilization of LTC, the most common type of PA was “walking+exercise” (53.2%), followed by “walking” (26.6%), no PA (10.3%), and “exercise” (9.9%). Individuals with utilization of LTC did no PA most often (39.3%), followed by “walking” (32.8%), “walking+exercise” (20.8%), and “exercise” (7.2%).

**Table 1:** Study sample characteristics stratified by utilization of long-term care and type of physical activity at t_2_ (*n* = 4012)

		Total number of individuals per category		Individuals without long-term care *n* = 3250 (81.0%)					Individuals with utilization of long-term care *n* = 762 (19.0%)				
				Total	No physical activity^a^ *n* = 334 (10.3%)	Walking only^b^ *n* = 864 (26.6%)	Exercise only^c^ *n* = 323 (9.9%)	Walking + exercise^d^ *n* = 1727 (53.2%)	Total	No physical activity^a^ *n* = 296 (39.3%)	Walking only^b^ *n* = 247 (32.8%)	Exercise only^c^ *n* = 54 (7.2%)	Walking + exercise^d^ *n* = 157 (20.8%)
Age in years	total	4002	75.0 (±6.6)	73.9 (±6.0)	76.1 (±6.3)	75.2 (±6.4)	73.6 (±6.1)	72.9 (±5.4)	79.5 (±7.3)	81.1 (±7.2)	79.9 (±7.3)	77.8 (±6.9)	76.5 (±6.5)
Sex	female	4002	2099 (52.5%)	1621 (49.9%)	178 (11.0%)	444 (27.4%)	166 (10.2%)	833 (51.4%)	478 (63.4%)	180 (37.7%)	158 (33.1%)	33 (6.9%)	107 (22.4%)
	male		1903 (47.6%)	1627 (50.1%)	156 (9.6%)	420 (25.8%)	157 (9.7%)	894 (55.0%)	276 (36.6%)	116 (42.0%)	89 (32.3%)	21 (7.6%)	50 (18.1%)
Education in years	total	4000	11.3 (±2.6)	11.4 (±2.6)	11.0 (±2.5)	11.0 (±2.9)	11.8 (±2.9)	11.6 (±2.6)	10.8 (±2.4)	10.5 (±2.2)	10.7 (±2.4)	11.2 (±2.7)	11.3 (±2.5)
Living arrangement	alone	4002	1183 (29.6%)	850 (26.2%)	79 (9.3%)	255 (30.0%)	89 (10.5%)	427 (50.2%)	333 (44.2%)	120 (36.0%)	121 (36.3%)	20 (6.0%)	72 (21.6%)
	not alone		2819 (70.5%)	2398 (73.8%)	255 (10.6%)	609 (25.4%)	234 (9.8%)	1300 (54.2%)	421 (55.8%)	176 (41.8%)	126 (29.9%)	34 (8.1%)	85 (20.2%)
Income	sufficient	3968	3303 (83.2%)	2744 (85.1%)	271 (9.9%)	715 (26.1%)	277 (10.1%)	1481 (54.0%)	559 (75.0%)	205 (36.7%)	195 (34.9%)	43 (7.7%)	116 (20.8%)
	scarce/not sufficient		665 (16.8%)	479 (14.7%)	60 (12.5%)	139 (29.0%)	43 (9.0%)	237 (49.5%)	186 (25.0%)	85 (45.7%)	49 (26.3%)	11 (5.9%)	41 (22.0%)
Falls within last year	≥ 1 fall	3997	575 (14.4%)	331 (10.2%)	50 (15.1%)	103 (31.1%)	37 (11.2%)	141 (42.6%)	244 (32.4%)	113 (46.3%)	65 (26.6%)	17 (7.0%)	49 (20.1%)
	no falls/unknown		3422 (85.6%)	2913 (89.8%)	283 (9.7%)	761 (26.1%)	285 (9.8%)	1584 (54.4%)	509 (67.6%)	183 (36.0%)	181 (35.6%)	37 (7.3%)	108 (21.2%)
BMI	total	3949	27.1 (±4.5)	27.0 (±4.3)	28.5 (±5.2)	27.4 (±15.1)	27.3 (±4.4)	26.5 (±3.9)	27.3 (±5.2)	28.2 (±6.0)	27.0 (±4.6)	27.3 (±5.0)	26.4 (±4.4)
Multimorbidity in no. of chronic conditions	total	3976	2.3 (±1.5)	2.0 (±1.4)	2.4 (±1.4)	2.3 (±1.4)	2.2 (±1.4)	1.8 (±1.3)	3.5 (±1.7)	3.7 (±1.7)	3.6 (±1.7)	2.9 (±1.5)	3.1 (±1.7)
Disability score (HAQ-DI)	total	4000	0.3 (±0.6)	0.2 (±0.3)	0.4 (±0.5)	0.2 (±0.3)	0.2 (±0.3)	0.1 (±0.2)	1.1 (±0.8)	1.5 (±0.9)	0.9 (±0.7)	0.9 (±0.7)	0.7 (±0.6)

*HAQ-DI* Health Assessment Questionnaire Disability Index

Data presented as n (%)/ mean (± standard deviation) | any discrepancies to total N due to missing values | any discrepancies in percentages due to rounding

^a^ “no physical activity” = “no or low walking (≤ 15 min/weekday)” + “no or low exercise” (see Additional file 1 for more information)

^b^ “walking only” = “high or moderate walking (> 15 min/weekday)” + “no or low exercise”

^c^ “exercise only” = “high or moderate exercise” + “no or low walking (≤ 15 min/weekday)”

^d^ “walking+exercise” = “high or moderate walking (> 15 min/weekday)” + “high or moderate exercise”

Generally, individuals with no PA were older, had less education, and had a higher BMI, higher multimorbidity, and a higher disability score. Within individuals without utilization of LTC, those with “walking+exercise” (the most frequently completed type of PA) as compared to the other types of PA were the youngest (72.9; SD: 5.4); they had the second most years of education (11.6; SD: 2.6) after those who did “walking” (11.8; SD: 2.9), the lowest BMI (26.5, SD: 3.9), the lowest multimorbidity (1.8; SD: 1.3), and the lowest disability score (0.1; SD: 0.2). Looking at individuals with utilization of LTC, those with no PA (the most frequently completed type of PA) were the oldest (81.1; SD: 7.2); they had the fewest years of education (10.5; SD: 2.2), the highest BMI (28.2; 6.0), the highest multimorbidity (3.7; SD: 1.7), and the highest disability score (1.5; SD: 0.9) as compared to those with other types of PA. The sample at t_1_ showed similar characteristics (see Table 6 in Appendix 1).

Dropouts between t_1_ and t_2_ with information about utilization of LTC and PA status (*n* = 248) used LTC (39.1%) more often, were older (81.2 years (SD: 6.4)), lived alone (55.7%) more frequently, had higher multimorbidity (3.0; SD: 1.6), and a higher disability score (0.8; SD: 0.8) than non-dropouts.

### Association of physical activity with utilization of long-term care

Table 2 displays the association of distinct types of PA with utilization of LTC in all observations (*n* = 4812) and in observations without disability (SA1, *n* = 3504). Compared to no PA, all types of PA were associated with reduced odds of utilization of LTC in the main analysis and SA1. “Walking” reduced the odds of utilization of LTC by 27% (OR: 0.73; CI: 0.56–0.95) and “exercise” reduced it by 44% (OR: 0.56; CI: 0.38–0.81). The combination of “walking+exercise” achieved the highest reduction, with a 48% decrease (OR: 0.52; CI: 0.39–0.70). The covariate being “female” increased the odds of utilization of LTC by 41% (OR: 1.41; CI: 1.12–1.76). Other covariates that increased the odds of utilization of LTC to a statistically significant degree were older age (OR: 1.04; CI: 1.03–1.06), higher education (OR: 1.05; CI: 1.00–1.09), living alone (OR: 1.56; CI: 1.26–1.93), falls (1.46; CI: 1.14–1.89), higher multimorbidity (OR: 1.30; CI: 1.22–1.39), and a higher disability score (OR: 6.85; CI: 6.85–10.78). SA1 and SA2 (Table 7 in Appendix 2) confirmed those results.

**Table 2:** Association of physical activity with utilization of long-term care – GEE logistic model

	Main analysis: LTC vs. no LTC in all observations^a^			Sensitivity analysis: LTC vs. no LTC in observations without disability^b^		
	Odds ratio	95% confidence interval	*p* value	Odds ratio	95% confidence interval	*p* value
Physical activity (ref.: no physical activity)^c^						
Walking only	0.73	[0.56; 0.95]	**0.0208**	0.78	[0.51; 1.19]	0.2463
Exercise only	0.56	[0.38; 0.81]	**0.0022**	0.55	[0.31; 0.98]	**0.0417**
Walking + exercise	0.52	[0.39; 0.70]	**< 0.0001**	0.44	[0.29; 0.66]	**0.0001**
**Adjusted for:**						
Sex (ref.: male)	1.41	[1.12; 1.76]	**0.0031**	1.49	[1.08; 2.06]	**0.0157**
Age in years	1.04	[1.03; 1.06]	**< 0.0001**	1.07	[1.04; 1.10]	**< 0.0001**
Education in years	1.05	[1.00; 1.09]	**0.0296**	1.08	[1.02; 1.13]	**0.0063**
Living arrangement (ref: not alone)	1.56	[1.26; 1.93]	**< 0.0001**	1.80	[1.32; 2.45]	**0.0002**
Income (ref: sufficient)	1.16	[0.91; 1.49]	0.2375	1.26	[0.87; 1.82]	0.2280
BMI	0.98	[0.96; 1.00]	0.0666	0.98	[0.95; 1.02]	0.3883
Falls (ref.: no falls/unknown)	1.46	[1.14; 1.89]	**0.0031**	1.99	[1.38; 2.89]	**0.0003**
Multimorbidity in no. of chronic conditions	1.30	[1.22; 1.39]	**< 0.0001**	1.46	[1.32; 1.61]	**< 0.0001**
Disability score (HAQ-DI)	8.60	[6.85; 10.78]	**< 0.0001**	/	/	/

*GEE* Generalized estimating equation | *LTC* Long-term care | *HAQ-DI* Health Assessment Questionnaire Disability Index

Bold numbers: significant at *p* ≤ 0.05

Sample for generalized estimating equation (*n* = 4812): sum of t_1_ (*n* = 800) and t_2_ sample (*n* = 4012)

^a^ Model includes all observations (*n* = 4812); observations stratified by either utilization of long-term care (*n* = 950) or no long-term care (*n* = 3862)

^b^ Model includes all observations without disability (HAQ-DI < 0.5) (*n* = 3504); observations stratified by either utilization of long-term care (*n* = 244) or no long-term care (*n* = 3260)

^c^ Categories of physical activity defined as: “no physical activity” = “no or low walking (≤15 mins/weekday)” + “no or low exercise” (see Additional file 1 for more information) | “walking only” = “high or moderate walking (>15mins/weekday)” + “no or low exercise” | “exercise only” = “high or moderate exercise” + “no or low walking (≤15 mins/weekday)” | “walking+exercise” = “high or moderate walking (>15mins/weekday)” + “high or moderate exercise”

### Sex-specific association of physical activity with utilization of long-term care

Tables 3 and 4 illustrate the association of PA with utilization of LTC in females, and in males, respectively. As in the entire cohort, each type of PA reduced the odds of utilization of LTC when compared to no PA. Statistically significant covariates in both females and males were older age, higher multimorbidity, and a higher disability score. “Walking” reduced the odds of utilization of LTC by 28% in females (OR: 0.72; CI: 0.51–1.02) and by 24% in males (OR: 0.76; CI: 0.50–1.15). In females, “exercise” reduced the odds of utilization of LTC by 42% (OR: 0.58; CI: 0.35–0.94), and in males it reduced the odds by 48% (0.52; CI: 0.30–0.93). “Walking+exercise” reduced the odds of utilization of LTC by 38% (OR: 0.62; CI: 0.44–0.89) in females and by 59% (OR: 0.41; CI: 0.26–0.65) in males. Looking at SA1, no type of PA had a statistically significant association with utilization of LTC in females. In contrast, among males the association of all types of PA remained statistically significant and was even stronger than in the main analysis.

**Table 3:** Association of physical activity with utilization of long-term care in females – GEE logistic model

	Main analysis: LTC vs. no LTC in all observations^a^			Sensitivity analysis: LTC vs. no LTC in observations without disability^b^		
	Odds ratio	95% confidence interval	*p* value	Odds ratio	95% confidence interval	*p* value
Physical activity (ref.: no physical activity)^c^						
Walking only	0.72	[0.51; 1.02]	0.0620	1.24	[0.60; 2.56]	0.5550
Exercise only	0.58	[0.35; 0.94]	**0.0288**	0.82	[0.32; 2.07]	0.6666
Walking + exercise	0.62	[0.44; 0.89]	**0.0094**	0.95	[0.49; 1.86]	0.8906
**Adjusted for:**						
Age in years	1.05	[1.02; 1.07]	**< 0.0001**	1.08	[1.04; 1.12]	**< 0.0001**
Education in years	1.04	[0.98; 1.11]	0.1773	1.07	[0.99; 1.16]	0.0895
Living arrangements (ref: not alone)	1.20	[0.92; 1.57]	0.1718	1.49	[1.00; 2.22]	0.0505
Income (ref: sufficient)	1.14	[0.83; 1.56]	0.4128	1.16	[0.65; 2.05]	0.6162
BMI	0.99	[0.96; 1.01]	0.3857	1.00	[0.95; 1.04]	0.9063
Falls (ref.: no falls/unknown)	1.49	[1.09; 2.05]	**0.0128**	2.16	[1.31; 3.59]	**0.0028**
Multimorbidity in no. of chronic conditions	1.26	[1.15; 1.37]	**< 0.0001**	1.43	[1.24; 1.66]	**< 0.0001**
Disability score (HAQ-DI)	9.45	[7.02; 12.71]	**< 0.0001**	/	/	/

*GEE* Generalized estimating equation | *LTC* Long-term care | *HAQ-DI* Health Assessment Questionnaire Disability Index

Bold numbers: significant at *p* ≤ 0.05

Sample for generalized estimating equation (*n* = 2499): sum of t_1_ (*n* = 395) and t_2_ sample (*n* = 2104)

^a^ Model includes all observations (*n* = 2499); observations stratified by either utilization of long-term care (*n* = 605) or no long-term care (*n* = 1894)

^b^ Model includes all observations without disability (HAQ-DI < 0.5) (*n* = 1669); observations stratified by either utilization of long-term care (*n* = 136) or no long-term care (*n* = 1533)

^c^ Categories of physical activity defined as: “no physical activity” = “no or low walking (≤15 mins/weekday)” + “no or low exercise” (see Additional file 1 for more information) | “walking only” = “high or moderate walking (>15mins/weekday)” + “no or low exercise” | “exercise only” = “high or moderate exercise” + “no or low walking (≤15 mins/weekday)” | “walking+exercise” = “high or moderate walking (>15mins/weekday)” + “high or moderate exercise”

**Table 4:** Association of physical activity with utilization of long-term care in males – GEE logistic model

	Main analysis: LTC vs. no LTC in all observations^a^			Sensitivity analysis: LTC vs. no LTC in observations without disability^b^		
	Odds ratio	95% confidence interval	*p* value	Odds ratio	95% confidence interval	*p* value
Physical activity (ref.: no physical activity)^c^						
Walking only	0.76	[0.50; 1.15]	0.1930	0.49	[0.27; 0.86]	**0.0140**
Exercise only	0.52	[0.30; 0.93]	**0.0274**	0.34	[0.15; 0.77]	**0.0098**
Walking + exercise	0.41	[0.26; 0.65]	**0.0002**	0.19	[0.10; 0.35]	**< 0.0001**
**Adjusted for:**						
Age in years	1.04	[1.01; 1.07]	**0.0033**	1.06	[1.02; 1.10]	**0.0026**
Education in years	1.06	[1.00; 1.12]	0.0611	1.08	[1.00; 1.16]	**0.0390**
Living arrangements (ref: not alone)	2.64	[1.88; 3.72]	**< 0.0001**	2.55	[1.57; 4.06]	0.0001
Income (ref: sufficient)	1.24	[0.83; 1.85]	0.2852	1.41	[0.79; 2.51]	0.2414
BMI	0.96	[0.92; 1.00]	**0.0458**	0.96	[0.90; 1.02]	0.1847
Falls (ref.: no falls/unknown)	1.42	[0.94; 2.16]	0.0961	1.77	[0.94; 3.33]	0.0761
Multimorbidity in no. of chronic conditions	1.38	[1.24; 1.53]	**< 0.0001**	1.52	[1.32; 1.75]	**< 0.0001**
Disability score (HAQ-DI)	7.78	[5.53; 10.94]	**< 0.0001**	/	/	/

*GEE* Generalized estimating equation | *LTC* Long-term care | *HAQ-DI* Health Assessment Questionnaire Disability Index

Bold numbers: significant at *p* ≤ 0.05

Sample for generalized estimating equation (*n* = 2313): sum of t_1_ (*n* = 405) and t_2_ sample (*n* = 1908)

^a^ Model includes all observations (*n* = 2313); observations stratified by either utilization of long-term care (*n* = 345) or no long-term care (*n* = 1968)

^b^ Model includes all observations without disability (HAQ-DI < 0.5) (*n* = 1835); observations stratified by either utilization of long-term care (*n* = 108) or no long-term care (*n* = 1727)

^c^ Categories of physical activity defined as: “no physical activity” = “no or low walking (≤15 mins/weekday)” + “no or low exercise” (see Additional file 1 for more information) | “walking only” = “high or moderate walking (>15mins/weekday)” + “no or low exercise” | “exercise only” = “high or moderate exercise” + “no or low walking (≤15 mins/weekday)” | “walking+exercise” = “high or moderate walking (>15mins/weekday)” + “high or moderate exercise”

Tests of the interaction terms “types of PA*sex” (references: no PA, male) in the main analysis resulted in the following: “walking*female” (OR: 0.85; CI: 0.50–1.46), “exercise*female” (OR: 1.01; CI: 0.48–2.13), “walking*exercise” (OR: 1.31; CI: 0.75–2.28). Tests of the interaction terms in SA1 resulted in the following: “walking*female” (OR: 2.74; CI: 1.07–10.53), “exercise*female” (OR: 3.20; CI: 0.97–10.53), “walking*exercise” (OR: 5.03; CI: 1.99–12.70).

### Individuals meeting suggested minimum values in German National Physical Activity Recommendations for older adults

Table 5 displays the number of individuals who met the GNPAR at t_1_. Almost a fourth (24.5%, *n* = 196) completed the suggested minimum of ≥150 minutes/week of “moderate-intensity exercise”, whereas only 6.5% (*n* = 52) completed ≥75 minutes/week of “vigorous-intensity exercise”. A total of 6.4% (*n* = 51) did strength training more than twice a week. For all types of PA, the proportion of individuals without utilization of LTC who met the GNPAR was higher than that of individuals with utilization of LTC. Group differences between individuals with and without utilization of LTC in relation to “moderate-intensity exercise” and “moderate- or vigorous-intensity exercise” were statistically significant.

**Table 5:** Individuals meeting suggested minimum values in German National Physical Activity Recommendations for older adults at t_1_

	Total *n* = 800	No long-term care *n* = 612 (76.5%)	Long-term care *n* = 188 (23.5%)
Moderate-intensity exercise ≥ 150 min/week ^a^	196 (24.5%)	174 (28.4%)	22 (11.2%)
Vigorous-intensity exercise ≥ 75 min/week	52 (6.5%)	52 (8.5%)	0 (0.0%)
Moderate-intensity ≥ 150 min/week or vigorous-intensity exercise ≥ 75 min/week^a^	222 (27.7%)	200 (32.7%)	22 (11.7%)
Strength training ≥ 2 times/week	51 (6.4%)	45 (7.4%)	6 (3.2%)

Multiple answers possible

Number and % of individuals completing each PA type were calculated based on the following:

1. Frequency: participants were asked “How often did you spend time doing [X] within the last 7 days?” per category (examples were given to specify the categories)

2. Time: if participants chose a category other than “not applicable/0 days”, participants were asked: “How many hours have you spent on average doing [X] within the last 7 days?” (not applied for strength training)

3. Amount in min/week (lower bounds): frequency * time (not applied for strength training)

4. Number/% of individuals: all individuals with ≥150 min/week moderate-intensity exercise/ ≥ 75 min/week vigorous-intensity exercise/ ≥ 2 times/week strength training were counted

^a^ *p* ≤ 0.005 | group differences “no long-term care” vs. “long-term care” and exercise intensity, analysis through chi^2^-tests

## Discussion

Our study is among the first to investigate the association of various types of PA with utilization of LTC in community-dwelling older adults in Germany. Compared to physically inactive individuals, those being physically active were less likely to utilize LTC. The combination of “walking+exercise” showed the strongest association with non-utilization of LTC in the entire cohort and in males. In contrast, among females, “exercise” had the strongest association with non-utilization of LTC. The proportion of individuals who completed the minimum values suggested by the GNPAR was higher among those without utilization of LTC than among those with utilization of LTC. In both individuals with and without utilization of LTC, the minimum values for “moderate-intensity exercise” were completed more often than the minimum values for “vigorous-intensity exercise” or “strength training”.

Our results suggest that being physically active is associated with reduced odds of utilization of LTC. Due to the lack of studies on the association of the outcome “utilization of LTC” with PA, and given that utilization of LTC is a complex construct, influenced by various determinants [18, 34], comparison with current evidence is limited. Until now, research mainly focused on the impact of PA on need factors (e. g., physical or cognitive problems) leading to utilization of health care services [32]. Evidence has demonstrated that various types of PA positively influence need factors [15, 54, 55]. This supports our results, although further studies with similar outcomes are needed to allow comparison of effects across studies.

Our results contribute to the evidence about the association of various types of PA with utilization of LTC in a subgroup of interest to policy-makers (i. e., older adults) [35]. WHO’s “Guidelines on physical activity and sedentary behaviour” [5] strongly recommend that older adults do varied multicomponent PA addressing “functional balance and strength training at moderate or higher intensity” at least three times per week. Our results show that the combination of “walking+exercise” had a stronger association with non-utilization of LTC than “walking” or “exercise”. Thus, that combination should be promoted more than “exercise” or “walking” alone in future PA programs for older adults. Still, to create evidence-based PA recommendations for community-dwelling older adults, specific subtypes (e. g., swimming, cycling), duration, and frequency of PA must be investigated in future longitudinal studies.

Comparing females with males based on the sex-stratified analyses, the association of “exercise” and “walking+exercise” with non-utilization of LTC was higher in males than in females. Older males prefer more vigorous exercise than females do [9] and the GNPAR assume that “increased energy expenditure at higher intensities ‘counts’ more” [35]. Therefore, we presume that males’ exercise was of higher intensity than females’ and thus resulted in the larger effect on non-utilization of LTC. However, it must be considered that the interaction terms “types of PA*sex” showed statistically significant sex differences solely in the cohort without disability. Thus, we recommend further studies investigating sex-specific effects of PA intensities on utilization of LTC.

While the association of PA with non-utilization of LTC was even stronger in males without disability compared to the entire male cohort, for females no corresponding association was found. A possible explanation might be gender differences in household PA. Due to persistent social and cultural norms, older females complete most household chores. Murphy et al. [10] analyzed total moderate- to vigorous-intensity exercise in both sexes. After excluding domestic PA, the proportion of females meeting PA guidelines decreased, whereas in males it stayed almost equal [10]. As PA effects are curvilinear [35], i. e. PA in already physically active individuals has a lower impact than in inactive individuals, we suspect that the effect of leisure-time PA in our female cohort without disability is marginal [8]. However, one must consider that our group of females without disability was relatively small. Thus, further research regarding this finding is urgently needed.

With 27.7% following WHO’s recommendations for moderate to vigorous PA, our sample falls within the documented range for countries worldwide (20–60%) [5, 7, 8]. The Robert Koch Institute (RKI), Germany’s core institution for nationwide health monitoring [56], examined the German population (representative sample) ≥ 65 years in 2019/2020. It found that 38.2% were physically active ≥150 minutes/week [57]. In comparison, our cohort was less active. The lower proportion meeting the GNPAR in our cohort could be explained by the lack of detailed differentiation of PA intensities by the RKI, resulting in this German cohort probably also including some types of low-intensity exercise (e. g., riding a bike at low speed), or including a lower proportion of individuals with utilization of LTC than ours. In our cohort, almost a fourth (24.5%) met the suggested minimum of ≥150 minutes per week of “moderate-intensity exercise”, whereas only 6.5% met the minimum of ≥75 minutes/week of “vigorous-intensity exercise”. This aligns with previous research stating that in older age “moderate-intensity exercise” is done more often than “vigorous-intensity exercise” [7, 8]. As falls in older age are one of the leading causes of transitions to utilization of LTC, they should be prevented through verifiably effective interventions, such as strength training [5, 35, 58]. In our cohort, only 6.4% did strength training more than twice a week, whereas in the RKI’s cohort, 30.3% did strength training at least twice a week [57]. This large discrepancy may be due the KORA questionnaire using the time frame “> 2 times/week” instead of “≥ 2 times/week”, whereas the RKI questionnaire used an open question (“How often did you spend time doing strength-training in a typical week?”). Thus, KORA assessments probably underestimated the proportion of individuals meeting the GNPAR.

We detected that the proportion of individuals meeting the GNPAR was much higher in individuals without utilization of LTC than in individuals with utilization of LTC. Also, considering our finding that individuals with utilization of LTC were mostly physically inactive and that, according to Ruetten et al. [35], the “greatest health benefits occur when individuals who were entirely physically inactive become somewhat more active”, there is a clear need to encourage this group to do some PA rather than none at all.

### Limitations and strengths

Our results must be interpreted with some caveats. First, we did not aim to assess causal relationships of PA with utilization of LTC. To investigate causal relationships, other study designs are needed. Our findings do suggest, however, that promoting PA in old age is associated with reduced odds of utilization of LTC.

Another limitation is the relatively small size of groups of individuals with LTC per subcategory of PA, which renders corresponding results less reliable. Still, as up to now there is no comparable analysis regarding this topic, our study contributes relevant evidence. Furthermore, our questionnaire-based study of community-dwelling older adults is relatively large in comparison to other representative regional cohort studies addressing PA in older adults and included relatively even proportions of females and males [8].

As mentioned above, we may have underestimated the proportion of individuals meeting the GNPAR. Due to the assessment of time frames (e. g., 1–2 hours/week) rather than estimated mean duration/day, we could not calculate the exact mean duration/week for each type of PA. However, we took the lower bound of each time frame (e. g., 1 hour/week) to avoid the common problem of overreporting PA through self-reports [8, 59].

Our study has several strengths that improve upon limitations of previous studies of PA measurements and evaluations of PA’s effects. First, standardized assessment and utilization of quality management in KORA studies (e. g., plausibility checks of participants’ answers by independent interviewers and data analysts) ensured high data quality [19]. Moreover, we based our approach to detecting relevant covariates and controlling for them on factors explored in current literature on this topic. Additionally, the GEE logistic model allowed us to consider intra-subject correlation in repeated measurements. Furthermore, the detailed assessment of relevant types of PA in old age (walking vs. exercise) addresses a highly relevant topic and therefore reduces the current gap in evidence about the effect of various types of PA on older adults [60, 61]. Thus, our findings can help to create target-oriented, subtype-specific PA recommendations, as well as PA promotion programs for community-dwelling older adults.

## Conclusions

Our results demonstrate an association between PA and non-utilization of LTC in community-dwelling older adults with sex-specific and disability-related particularities regarding distinct types of PA. Furthermore, they illustrate that the GNPAR are rarely met by older adults with and without utilization of LTC. To minimize or even partially prevent the public health issue of an increasing need for and thus higher utilization of LTC, policy-makers and health care workers should develop target-oriented PA promotion programs. For those programs, consideration of accessible and sustainable environments, as well as the target-groups’ needs, is indispensable for reaching this vulnerable group and fostering beneficial PA behaviors.

### Supplementary Information

**Additional file 1.** Title: Categorization of exercise. Description of data: Illustration explaining the transformation of the variable “exercise”.

**Additional file 2.** Title: Extract of questionnaire assessing type, frequency, and duration of physical activity at t_2_. Description of data: Questions used to assess types, frequency, and duration of physical activity at t_2_.

**Additional file 3.** Title: STROBE-checklist. Description of data: Checklist to determine quality, structure, and content of study.

## Data Availability

The data are subject to national data protection laws and restrictions were imposed by the Ethics Committee to ensure data privacy of the study participants. Therefore, data cannot be made freely available in a public repository. However, in reasonable cases data can be requested through individual project agreements via the KORA-PASST tool under https://epi.helmholtz-muenchen.de/.

## Appendix 1

**Table 6:** Study sample characteristics stratified by utilization of long-term care and type of physical activity at t_1_ (*n* = 800)

		Total number of individuals per category		Individuals without long-term care *n* = 612 (76.4%)					Individuals with utilization of long-term care *n* = 188 (23.5%)				
				Total	No physical activity *n* = 63 (10.3%)	Walking only *n* = 184 (30.1%)	Exercise only *n* = 58 (9.5%)	Walking + exercise *n* = 307 (50.2%)	Total	No physical activity *n* = 70 (37.6%)	Walking only *n* = 56 (30.1%)	Exercise only *n* = 18 (9.7%)	Walking + exercise *n* = 42 (22.6%)
Age in years	total	798	78.3 (±6.4)	77.1 (±6.0)	79.3 (±6.9)	78.9 (±5.6)	77.3 (±5.7)	75.5 (±5.6)	82.3 (±6.0)	85.0 (±4.7)	81.4 (±6.3)	78.8 (±5.5)	80.5 (±5.9)
Sex	female	798	394 (49.4%)	272 (44.4%)	22 (8.1%)	88 (32.4%)	25 (9.2%)	137 (50.4%)	122 (65.6%)	51 (41.8%)	36 (29.5%)	10 (8.2%)	25 (20.5%)
	male		404 (50.6%)	340 (55.6%)	41 (12.1%)	96 (28.2%)	33 (9.7%)	170 (50.0%)	64 (34.4%)	19 (29.7%)	20 (31.3%)	8 (12.5%)	17 (26.6%)
Education in years	total	798	10.9 (±2.6)	11.0 (±2.6)	11.3 (±2.7)	10.8 (±2.6)	11.2 (±2.5)	11.0 (±2.5)	10.5 (±2.5)	10.2 (±2.6)	10.5 (±2.6)	10.2 (±2.2)	11.1 (±2.5)
Living arrangement	alone	798	281 (35.2%)	183 (29.9%)	13 (7.1%)	65 (35.5%)	21 (11.5%)	84 (45.9%)	98 (52.7%)	37 (37.8%)	27 (27.6%)	8 (8.2%)	26 (26.5%)
	not alone		517 (64.8%)	429 (70.1%)	50 (8.2%)	119 (19.4%)	37 (6.1%)	223 (52.0%)	88 (47.3%)	33 (37.5%)	29 (33.0%)	10 (11.4%)	16 (18.2%)
Income	sufficient	794	632 (79.6%)	502 (79.4%)	49 (9.8%)	149 (29.7%)	48 (9.6%)	256 (51.0%)	130 (20.6%)	47 (36.2%)	41 (31.5%)	12 (9.2%)	30 (23.1%)
	scarce/not sufficient		162 (20.4%)	108 (66.7%)	13 (12.0%)	35 (32.4%)	10 (9.3%)	50 (46.3%)	54 (33.3%)	21 (38.9%)	15 (27.8%)	6 (11.1%)	12 (22.2%)
Falls within last year	≥ 1 fall	798	141 (17.7%)	77 (12.6%)	13 (16.9%)	31 (40.3%)	7 (9.1%)	26 (33.8%)	64 (45.4%)	29 (45.3%)	17 (26.6%)	7 (10.9%)	11 (17.2%)
	no falls/ unknown		657 (82.3%)	535 (87.4%)	50 (9.4%)	153 (28.6%)	51 (9.5%)	281 (52.5%)	122 (65.6%)	41 (33.6%)	39 (32.0%)	11 (9.0%)	31 (25.4%)
BMI	total	791	28.1 (±4.2)	27.9 (±3.9)	27.9 (±3.5)	28.4 (±4.3)	28.5 (±4.3)	27.5 (±3.5)	28.7 (±5.2)	28.9 (±5.6)	28.7 (±5.2)	30.8 (±5.6)	27.3 (±4.1)
Multimorbidity in no. of chronic conditions	total	789	2.5 (±1.5)	2.3 (±1.4)	2.6 (±1.6)	2.5 (±1.5)	2.2 (±1.5)	2.2 (±1.3)	3.3 (±1.6)	3.7 (±1.7)	3.3 (±1.5)	2.5 (±1.2)	2.9 (±1.6)
Disability score (HAQ-DI)	total	798	0.5 (±0.7)	0.3 (±0.4)	0.5 (±0.6)	0.3 (±0.4)	0.3 (±0.5)	0.2 (±0.3)	1.2 (±0.8)	1.7 (±0.8)	0.9 (±0.7)	1.2 (±1.0)	0.6 (±0.5)

Data presented as n (%)/ mean (± standard deviation) | any discrepancies to total N due to missing values | any discrepancies in percentages due to rounding

*HAQ-DI* Health Assessment Questionnaire Disability Index

^a^ “no physical activity” = “no or low walking (≤ 15 min/weekday)” + “no or low exercise” (see Additional file 1 for more information)

^b^ “walking only” = “high or moderate walking (> 15min/weekday)” + “no or low exercise”

^c^ “exercise only” = “high or moderate exercise” + “no or low walking (≤ 15 min/weekday)”

^d^ “walking+exercise” = “high or moderate walking (> 15min/weekday)” + “high or moderate exercise”

## Appendix 2

**Table 7:** Association of physical activity with utilization of long-term care excluding observations with missing values in subdomains – GEE logistic model

	Main analysis: LTC vs. no LTC in all observations^a^			Sensitivity analysis: LTC vs. no LTC in observations without disability^b^		
	Odds ratio	95% confidence interval	*p* value	Odds ratio	95% confidence interval	*p* value
Physical activity (ref.: no physical activity)						
Walking only	0.72	[0.55; 0.94]	**0.0173**	0.78	[0.51; 1.19]	0.2488
Exercise only	0.56	[0.38; 0.81]	**0.0021**	0.55	[0.31; 0.98]	**0.0417**
Walking + exercise	0.52	[0.39; 0.69]	**<0.0001**	0.44	[0.29; 0.66]	**0.0001**
**Adjusted for:**						
Sex (ref.: male)	1.40	[1.11; 1.75]	**0.0039**	1.49	[1.08; 2.06]	**0.0154**
Age in years	1.04	[1.03; 1.06]	**<0.0001**	1.07	[1.04; 1.10]	**<0.0001**
Education in years	1.05	[1.00; 1.09]	**0.0312**	1.08	[1.02; 1.13]	**0.0063**
Living arrangement (ref: not alone)	1.56	[1.26; 1.93]	**<0.0001**	1.79	[1.32; 2.44]	**0.0002**
Income (ref: sufficient)	1.16	[0.90; 1.49]	0.2466	1.26	[0.87; 1.82]	0.2287
BMI	0.98	[0.96; 1.00]	0.0622	0.98	[0.95; 1.02]	0.3854
Falls (ref.: no falls/unknown)	1.47	[1.14; 1.89]	**0.0027**	1.99	[1.37; 2.89]	**0.0003**
Multimorbidity in no. of chronic conditions	1.30	[1.21; 1.39]	**<0.0001**	1.46	[1.32; 1.61]	**<0.0001**
Disability score (HAQ-DI)	8.53	[6.79; 10.71]	**<0.0001**	/	/	/

Bold numbers: significant at *p* ≤ 0.05

Sample for generalized estimating equation (*n* = 4799): sum of t_1_ (*n* = 798) and t_2_ sample (*n* = 4001)

*GEE* generalized estimating equation, *LTC* long-term care, *HAQ-DI*, Health Assessment Questionnaire Disability Index

^a^ Model includes all observations (*n* = 4799); observations stratified by either utilization of long-term care (*n* = 940) or no long-term care (*n* = 3859)

^b^ Model includes all observations without disability (HAQ-DI <0.5) (*n* = 3503); observations stratified by either utilization of long-term care (*n* = 244) or no long-term care (*n* = 3259)

^c^ Categories of physical activity defined as:

“no physical activity” = “no or low walking (≤15 mins/weekday)” + “no or low exercise” (see Additional file 1 for more information)

“walking only” = “high or moderate walking (>15mins/weekday)” + “no or low exercise”

“exercise only” = “high or moderate exercise” + “no or low walking (≤15 mins/weekday)”

“walking+exercise” = “high or moderate walking (>15mins/weekday)” + “high or moderate exercise”

## Abbreviations

BMI: Body Mass Index
CI: 95% confidence interval
GNPAR: German National Physical Activity Recommendations for older adults
LTC: Long-term care
OR: Odds ratio
PA: Physical activity
RKI: Robert Koch Institute
SA: Sensitivity analysis
SD: Standard deviation
WHO: World Health Organization
