# Supplementary material for: Association of physical activity with utilization of long-term care in community-dwelling older adults in Germany: results from the population-based KORA-Age observational study
Source: Int J Behav Nutr Phys Act. 2022 Aug 8;19:102. doi: 10.1186/s12966-022-01322-z (PMC9358813; doi:10.1186/s12966-022-01322-z)
Supplement: Supplementary file 1 — Additional file 1. Title: Categorization of exercise. Description of data: Illustration explaining the transformation of the variable “exercise”. [file 12966_2022_1322_MOESM1_ESM.pdf]

Additional file 1: Categorization of exercise

How often do you exercise during winter?	How often do you exercise during summer?			
	**1**	**2**	**3**	**4**
**1**	**high exercise**			
**2**		**moderate exercise**	**low exercise**	
**3**			**no exercise**	
**4**				

1 "regularly more than or equal to two hours per week"
2 "regularly more than or equal to one, but less than two hours per week"
3 "less than one hour per week"
4 "no exercise"

According to:

Karl FM, Tremmel M, Luzak A, Schulz H, Peters A, Meisinger C, et al. Direct healthcare costs associated with device assessed and self-reported physical activity: results from a cross-sectional population-based study. BMC Public Health. 2018;18:966. doi:10.1186/s12889-018-5906-7
