# Supplementary material for: Association of physical activity with utilization of long-term care in community-dwelling older adults in Germany: results from the population-based KORA-Age observational study
Source: Int J Behav Nutr Phys Act. 2022 Aug 8;19:102. doi: 10.1186/s12966-022-01322-z (PMC9358813; doi:10.1186/s12966-022-01322-z)
Supplement: Supplementary file 2 — Additional file 2. Title: Extract of questionnaire assessing type, frequency, and duration of physical activity at t2. Description of data: Questions used to assess types, frequency, and duration of physical activity at t2. [file 12966_2022_1322_MOESM2_ESM.pdf]

Additional file 2: Extract of questionnaire assessing type, frequency, and duration of physical activity at t_2

**Walking**	
How often did you spend time walking outside (e. g., to relax, to go shopping, to go to work, to walk with the dog) within the last 7 days?	□ not applicable/0 days<br>□ 1-2 days[a]<br>□ 3-4 days[a]<br>□ 5-7 days[a]
**Moderate-intensity exercise**	
How often did you spend time doing moderate-intensity exercise (e. g., dancing, gymnastics, riding a bike at moderate speed, moderate swimming, nordic walking or other similar activities) within the last 7 days?	□ not applicable/0 days*<br>□ 1-2 days[a]<br>□ 3-4 days[a]<br>□ 5-7 days[a]
**High-intensity exercise**	
How often did you spend time doing high-intensity exercise (e. g., running, intense swimming, riding a bike with high speed, hiking, aerobic, skiing or other similar activities) within the last 7 days?	□ not applicable/0 days<br>□ 1-2 days[a]<br>□ 3-4 days[a]<br>□ 5-7 days[a]
**Strength training**	
How often did you spend time doing strength-training (e. g., lifting weights, push-ups) within the last 7 days?	□ not applicable/0 days<br>□ 1-2 days[a]<br>□ 3-4 days[a]<br>□ 5-7 days[a]
**Filter question for [a]**	
How many hours on average did you spend doing [walking/moderate-intensity exercise/ high-intensity exercise/strength training] on the mentioned days?	□ less than 1 hour<br>□ more than 1, but less than 2 hours<br>□ 2 to 4 hours<br>□ more than 4 hours

Calculation of amount in min/week:

1. **Chosen number of days/week for frequency (i. e. the lowest value in the selected range)**:
   □ 0 days
   □ 1 day
   □ 3 days
   □ 5 days

2. **Chosen number of minutes/chosen frequency for time spent:**
   □ 30 minutes
   □ 60 minutes
   □ 120 minutes
   □ 270 minutes

3. **Amount in minutes/week:**
   frequency * time (not applied for strength training)
